# High information capacity DNA-based data storage with augmented encoding characters using degenerate bases

**DOI:** 10.1038/s41598-019-43105-w

**Published:** 2019-04-29

**Authors:** Yeongjae Choi, Taehoon Ryu, Amos C. Lee, Hansol Choi, Hansaem Lee, Jaejun Park, Suk-Heung Song, Seojoo Kim, Hyeli Kim, Wook Park, Sunghoon Kwon

**Affiliations:** 1 Department of Electrical and Computer Engineering, Seoul National University, 1, Gwanak-ro, Gwanak-gu, Seoul 08826 Republic of Korea; 2 Interdisciplinary Program for Bioengineering, Seoul National University, 1, Gwanak-ro, Gwanak-gu, Seoul 08826 Republic of Korea; 3 Department of Electronic Engineering, Kyung Hee University, Deongyeong-daero, Giheung-gu, Yongin-si, Gyeonggi-do, 17104 Republic of Korea; 4 Institute of Entrepreneurial Bio Convergence, Seoul National University, 1, Gwanak-ro, Gwanak-gu, Seoul 08826 Republic of Korea; 5 Seoul National University Hospital Biomedical Research Institute, Seoul National University Hospital, 101, Daehak-ro Jongno-gu, Seoul 03080 Republic of Korea; 6 Current Address: Celemics Inc., 131, Gasandigital 1-ro, Geumcheon-gu, Seoul 08506 Republic of Korea

**Keywords:** Biotechnology, Molecular biology

## Abstract

DNA-based data storage has emerged as a promising method to satisfy the exponentially increasing demand for information storage. However, practical implementation of DNA-based data storage remains a challenge because of the high cost of data writing through DNA synthesis. Here, we propose the use of degenerate bases as encoding characters in addition to A, C, G, and T, which augments the amount of data that can be stored per length of DNA sequence designed (information capacity) and lowering the amount of DNA synthesis per storing unit data. Using the proposed method, we experimentally achieved an information capacity of 3.37 bits/character. The demonstrated information capacity is more than twice when compared to the highest information capacity previously achieved. The proposed method can be integrated with synthetic technologies in the future to reduce the cost of DNA-based data storage by 50%.

## Introduction

The annual demand for digital data storage is expected to surpass the supply of silicon in 2040, assuming that all data are stored in flash memory for instant access^1^. Considering the massive accumulation of digital data, the development of alternative storage methods is essential. One alternative is DNA-based data storage, which converts the binary digital data of 0 and 1 into the quaternary encoding nucleotides A, C, G, and T, synthesizes the sequence, and stores the data^2,3^. This concept^2–10^ is attractive due to two main advantages: the high physical information density of petabytes of data per gram, and the durability as the storage lasts for centuries without energy input. Due to these advantages, DNA-based data storage is expected to supplement the increasing demand for digital data storage, especially for archival data that are not frequently accessed. Since DNA-based data storage was proposed, the major goal was to improve data to DNA encoding algorithms^9,10^ or error correction algorithms^4,6,7,9,10^ to reduce data error or loss considering the biochemical properties while handling DNA. These previous studies on encoding algorithms showed 100% reconstruction of the data from DNA while using library of 100 to 200nt length oligonucleotides. To correct the synthesis errors and recover the dropped data fragments during DNA amplification, the library of oligonucleotides that contains 1300 copies of each designed sequences were required^10^, with the developed algorithms.

The next step towards the practical use of DNA-based data storage is to reduce the cost of storing the data. The cost of DNA-based data storage is categorized into the cost of data writing through DNA synthesis and the cost of data reading through DNA sequencing. Among these two costs, the cost of data writing is predominant because it is tens of thousands times more expensive per unit DNA than that of reading. However, previous studies have shown that DNA can be put to practical use as a backup storage medium only when the cost of the data writing is approximately 100 times less^4^. There are several ways to solve this problem, such as development of cheaper DNA synthesis methods or DNA encoding algorithms. But, the most simple and straightforward way is to maximize the amount of data that can be stored per length of DNA sequence that is designed (information capacity, bits/character, see details on the definition in Supplementary Note) and minimize the DNA synthesis, with current DNA-based data storage strategies. Previous methods have a theoretical information capacity limit of *log*_2_4, or 2.0 bits/character, because DNA comprises four encoding characters (A, C, G, T). For example, the highest information capacity that was reached experimentally, 1.57 bits/character in 1300 copies of each sequence, was demonstrated in Erlich *et al*.^10^. However, if additional encoding characters are introduced, the information capacity of *log*_2_(number of encoding characters) dramatically increases, further reducing the cost of DNA data storage.

Here, we propose and demonstrate the use of degenerate bases (combination of the four DNA bases that can be inserted at any base sites within a sequence)^11^ as additional encoding characters to exceed the theoretical information capacity limit of 2.0 bits/character. Degenerate bases are located in the DNA sequence when nucleotides are mixed at a specific position in the DNA sequence. For example, in the sequence ‘AWC’, ‘W’ indicates a combination of A and T; thus, two types of nucleotide variants exist in the pool of molecules: ‘AAC’ and ‘ATC’. In this article, by using eleven degenerate bases in addition to the four DNA characters, we experimentally achieve an information capacity of 3.37 bits/character within oligonucleotide library comprising hundreds of copies of each sequence. In other words, we store more data using less copies of each sequence, compared to the molecule number used in previous studies. As a result, we demonstrate that the DNA length needed to store the same amount of data was reduced by more than half compared to previous reports^3–6,9,10^. The proposed technology can be integrated with synthetic technologies in the future to reduce the cost of DNA-based data storage by 50%.

## Results

### Addition of degenerate bases to DNA-based data storage

**Figure 1:**
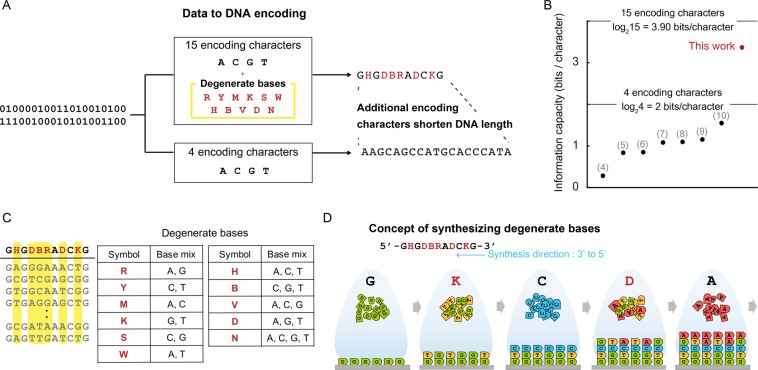
DNA-based data storage with addition of degenerate bases enables increased information capacity. (**A**) Binary data is encoded to DNA sequences comprising not only the 4 traditional encoding characters A, C, G, and T but also 11 additional degenerate bases. The length of encoded DNA is less than that of the four-character encoding method. (**B**) The theoretical information capacity limit is therefore increased from 2 bits/character to 3.9 bits/character. The dots in the graph describe the information capacity values in previous research, and the numbers indicate the corresponding reference. (**C**) A degenerate base represented by an encoding character describes a mixed pool of more than two types of nucleotides. (**D**) Degenerate bases can be generated by mixing the DNA phosphoramidites during the synthesis.

The conversion from a four to a fifteen character-based encoding system theoretically allows a maximum information capacity of 3.90(*log*_2_15) bits/character (previously 2.0 (*log*_2_4) bits/character) and shortens the length of DNA required to store an equivalent amount of data by approximately half (Fig. 1A). While previous research has increased the information capacity to near the theoretical limit by optimizing the data to DNA encoding algorithm, our approach increases the information capacity by increasing the theoretical limit (Fig. 1B). Also, while other researches compared in Fig. 1B used library of more than thousands copies of each oligonucleotide sequences, we achieved an empirical information capacity of more than 2bits per character within an oligonucleotide library comprising hundreds of copies for each sequence. The degenerative portion of the encoded sequence is incorporated by mixing the DNA phosphoramidites during the synthetic procedure^12^ and generating variants of the corresponding combinations of A, C, G and T (Fig. 1C,D). Ideally, for column-based^12^ and inkjet-based^13–15^ oligonucleotide synthesis, degenerate bases can be added without extra cost because the total amount of phosphoramidites used is the same(Supplementary Note). Also, current synthesis techniques synthesize more than billion molecules of oligonucleotides molecules per design, which are sufficient to generate variant pool for degenerate base. Therefore, the platform shortens the length of DNA to store the equivalent amount of data by approximately half, decreasing the expense for DNA synthesis (i.e. the data writing), if the appropriate synthesis method is applied.

### Structure and decoding result of the DNA-based data storage platform

**Figure 2:**
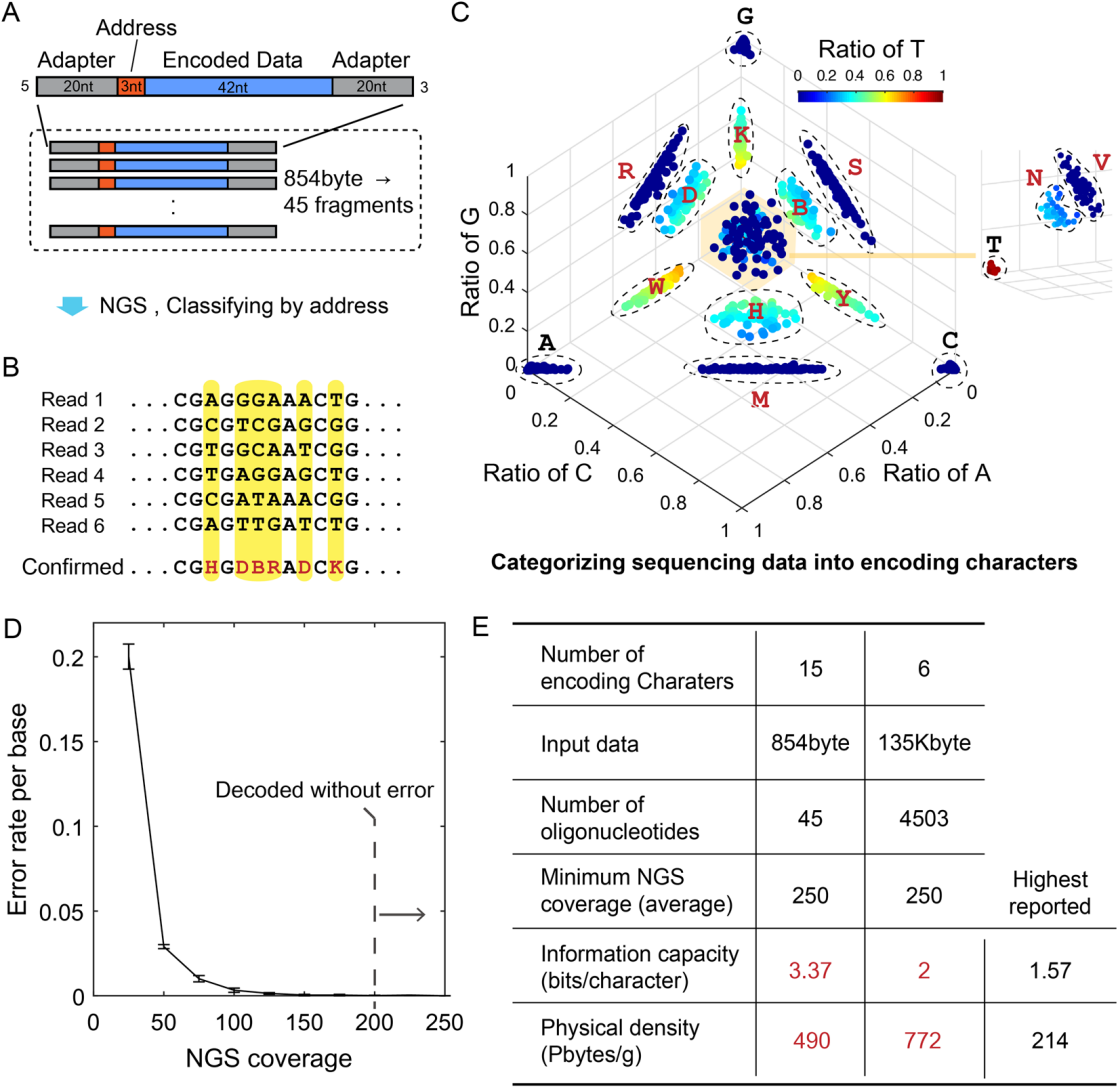
Structure and decoding result of the DNA-based data storage platform. We achieved the highest information capacity and physical density of DNA-based data storage. (**A**) Design structure of DNA fragments. (**B**) DNA fragments can be analyzed using NGS. After classification by address, degenerate bases can be decoded by examining the distribution of characters in the same position (yellow bar). (**C)** Degenerate bases can be determined from the scatter plot of the ratio of bases in the same position. (**D**) The error rate of determined DNA bases in specific average coverage of the total fragments. The standard deviations (s.d.) were obtained by repeating the random sampling 10 times. The error bars represent s.d. (**E**) Summary of the experimental results. The information capacity is calculated from the input information in bits divided by the number of encoding characters (excluding that of adapter sites). We compared the results of our work with those of Erlich and Zielinski^10^, who previously reported the highest information capacity and physical density using pooled oligo synthesis and high-throughput sequencing data. The physical density is the ratio of the number of bytes encoded to the weight of the DNA library used to decode the information.

We encoded an 854 bytes-text file to DNA sequences (Fig. 2, Fig. S1). The data were transformed into a series of three-character DNA codons, the sequence of which consists of three encoding characters. The last base in the sequence of the codons was designed to not be equivalent to the front-most base in the sequence of the next codon to avoid the generation of homopolymers of 4 nt or more (Table S1). The encoded information was divided into 42 nt fragments, and an address composed of 3 nt of non-degenerate bases (Table S2) was assigned to each fragment (Fig. 2A). Each fragment was supplemented with two adapters (20 nt each for the 5′ and 3′ end) for amplification and sequencing, and the entire fragment was 85 nt in length. From the design described, 45 DNA fragments were synthesized by the column-based oligonucleotide synthesizer without additional cost. Considering the number of bits encoded in the total nucleotide synthesis excluding the adapters, an information capacity of 3.37 bits/character was achieved experimentally, which is more than twice the highest reported value of 1.57 bits/character^10^. The information capacity demonstrated was lower than the theoretical maximum because the encoding efficiency was lowered to avoid homopolymer sequences and incorporate non-data address sequences for each fragment. The synthesized DNA library consisting of approximately 800 molecules was amplified by designed adapters and was sequenced by an Illumina MiniSeq. The raw data was filtered using the designed length and categorized by addresses. Then, the duplicated reads were removed and the distribution of A, C, G, and T in each position on the fragment was analysed (Fig. 2B). When we observed the ratio of A:C:G:T in the sequence analyzed at the same position using a scatter plot, the points were clustered into fifteen groups, eleven of which had an intermediate ratio of more than two bases considered degenerate bases (Fig. 2C). The other four that had a dominant ratio of a particular nucleotide were considered pure sequences. The intermediate ratio of the nucleotides analyzed was not consistently equivalent because the coupling efficiency during synthesis varies for each base, by type and position in the growing oligonucleotide^16–18^. To infer the degenerate bases, we introduced error elimination technique from the base calls. For example, if the base call of A and C is a determined as an error in the base calls, then G and T is the base intended from the design, and the encoding character inferenced is K. The errors identified while the base call analysis (Fig. 2B), which is the substitution, is known as about 1% of base calls. The probability distribution of these errors is directed towards zero so it can be distinguished from the base call corresponding to designed characters, even if the intermediate ratio of nucleotides is not known. We obtained the distribution of the calls in the sequencing reads and obtained the point that can distinguish the part that corresponds to the error. The classification method was to obtain the first inflection point from the distribution (Fig. S3). By comparing the decision points and the proportion of the nucleotide call from each character position, we inferred the intended bases, as well as the encoding character. Through this decoding process, we successfully recovered the original data from the raw next-generation sequencing (NGS) data. We also recovered the data in 10 of 10 cases when randomly down-sampled to the average coverage of 250x. If the average NGS coverage is lower than 250x, the error rate increases because the probability distribution of error overlaps with the distribution of intended bases (Fig. 2D).

To demonstrate the scalability of the introduced platform, we also stored 135.4 kB of data (Supplementary Fig. S2) in 4503 fragments of DNA using the pooled oligonucleotide synthesis method, which is high throughput. To manage the error^19^ and amplification bias that may occur when synthesizing and amplifying oligonucleotide pools with high complexity^20,21^, we added Reed-Solomon-based redundancy^9^ (Supplementary Note, Fig. S4). Even though only two degenerate bases, W and S, were used for this demonstration due to equipment constraints (Supplementary Note), an information capacity of 2.0 bits/character was achieved. We recovered the data in 10 of 10 cases when randomly down-sampling the average coverage to 250x (Fig. S5). This is higher than the minimum NGS coverage required for DNA-based data storage without degenerate bases, which is approximately 5x^8^. We summarized our experimental results in terms of the input data, number of oligonucleotides, minimum coverage, physical density, and information capacity (Fig. 2E). Physical density describes relation between molecule number used and data quantity (Supplementary Note), while information capacity describes that between designed character number and data quantity. Although we synthesized oligonucleotide variants in single designed fragments to incorporate the degenerate bases, fewer oligonucleotide molecules per fragment (hundreds) were sufficient to decode the data, than that in a previous report^10^. In this respect, we renewed the highest experimentally proven information capacity and physical density by compromising higher NGS coverage.

### Verification and cost projection of proposed platform via simulation

**Figure 3:**
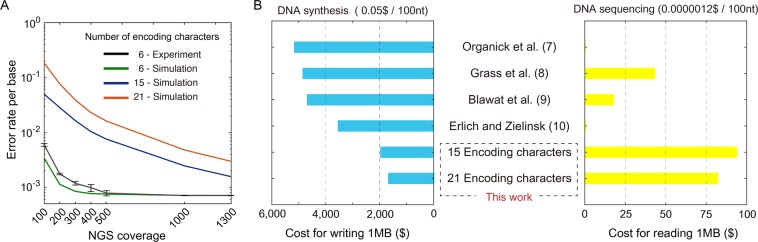
Error rate and cost for DNA-based data storage were analyzed. (**A**) The error rate per base pair according to the average NGS coverage over all fragments. The black line shows the experimental results, and the other three lines represent the Monte Carlo simulation results. For the experiment and simulation shown in green, we used A, C, G, T, W, and S for encoding. For the simulation shown in blue, we used A, C, G, T and all other degenerate bases. For the simulation shown in red, we used A, C, G, T, [R, Y, M, K, S, W – ratio of bases mixed of 3:7 and 7:3], H, V, D and N. The standard deviation of the experimental results were obtained by repeating the random sampling 5 times. The error bars represent the s.d. (**B**) The proposed platform is estimated to reduce the cost of DNA-based data storage by 50%. For the calculation, we assumed the cost of inkjet-based oligonucleotide pool synthesis reported by Erlich and Zielinski^10^. The cost of DNA sequencing was reported by K. Wetterstrand^22^. We used A, C, G, T and all other eleven degenerate bases as encoding characters. Additionally, we used A, C, G, T, [R, Y, M, K, S, W – ratio of bases mixed of 3:7 and 7:3], H, V, D and N as 21 encoding characters. The numbers indicate the corresponding reference. Details on the estimation method are described in the Supplementary Note.

In addition to the experimental results, we simulated the error rate of the platform in terms of NGS coverage for data recovery when various types of degenerate bases are used on a large scale. Because the call frequency of each base comprising the degenerate bases follows a binomial distribution (Fig. S6, Supplementary Note), the platform was modeled using Monte Carlo simulation. We simulated the error rate per base pair of the models by using various sets of degenerate bases (Fig. 3A) when fragments are represented unevenly due to amplification bias (Fig. S7). The assumed length of the fragment used in the simulation was 200 nt with a 20-nt adaptor at both ends, and the data was stored at 148 nt, except for the address of 12 nt. In the simulation, we also introduced additional characters specified by two nucleotides with different ratios (e.g., W1 for A:T = 3:7 and W2 for A:T = 7:3) and expanded the number of encoding characters to 21. The data show that the use of various types of degenerate bases increases the error rate but the error rate decreases with increasing NGS coverage. Given NGS coverage of 1300x or more, decoding 100 MB with 10% Reed-Solomon redundancy in all proposed cases can proceed without error. As a result, we achieved 2.67 bits/character when using 15 encoding characters and 3.05 bits/character when using 21 encoding characters. Although the platform requires high NGS coverage, the sequencing technology has a rapid speed of evolution, and the current state of art DNA sequencing cost per base (0.0000012$/100 nt)^10^ is approximately 50,000 times lower than the synthesis cost per base (~0.05$/100 nt, Supplementary Note)^22^ using inkjet-based oligonucleotide pool synthesizer. Moreover, since the cost of DNA sequencing is decreasing faster than the Moore’s law and faster than that of DNA synthesis, the price gap between the sequencing and synthesis will increase by orders, if the current trend continues^1,23^. When this cost is applied, even if the proposed platform has 2000x NGS coverage as an extreme case, the data reading cost will be less than 5% of the writing cost and less than 0.5%, which will be negligible, in five years (Fig. 3B). Assuming the inkjet-based oligonucleotide synthesizer is set for degenerate base synthesis, the proposed platform was estimated to reduce the cost of DNA-based data storage to $2052/1MB when using 15 encoding characters and $1795/1MB when using 21 encoding characters, which is approximately 50% of the previous minimum of $3555/1MB^10^ (Fig. 3B, Supplementary Note).

## Discussion

In this demonstration, by utilizing degenerate bases, the information capacity and physical density were more than doubled compared to those of previously reported DNA-based data storage platforms. In particular, as the information capacity increases, the platform shortens the length of DNA required to store an equivalent amount of data and decreases the total expense of data storage by half. The physical density will be increased with empirically in future researches, and studies that push the upper limit of physical density will be followed. Also, the introduced method reduces the time of synthesis, if an appropriate synthesis system is available. For example, the column-based oligonucleotide synthesizing technique that uses washing, deprotection steps which increases in proportion to the length of the oligonucleotides to be synthesized. Because we can shorten the synthesis length for storing the same amount of data, the time of synthesis will be decreased.

To realize what is simulated in this study in large scale data storage, further development in oligonucleotide synthesis will be necessary. First, an oligonucleotide pool synthesis setup can be used to increase the information capacity by incorporating all the degenerate bases in the encoding characters by addition of the nozzles. Second, if the synthesis setup can precisely control the ratio of the nucleotides consisting degenerate base is developed, even more encoding characters can be used. Currently, no method that precisely control the ratio has been reported to the best of our knowledge and the most relevant and latest researches report that incorporation rate of A, C, G, T is different, and it varies according to the location in the oligonucleotide^16–18^. With future research, if it is possible to optimize the platform for a large-scale experiment and to generate modified degenerate bases with non-equivalent ratios suggested in the simulation, the cost of the data writing in DNA-based data storage will dramatically decrease to the point where it can be practically implemented in real-world use. Ideally, if methods that can precisely control the ratio of the nucleotide in the degenerate base is developed, infinite number of encoding characters can be used. To decode this precisely, further research in inferring the character can be followed. Since the base call probability follows multinomial, the development of decoding methods would be possible. Additionally, if synthesis and sequencing methods for synthetic bases^24^ are developed, they can be used as other types of encoding characters. In addition to the development of these synthetic methods, reduction in the DNA amplification bias will improve the practical efficiency of the method. Together with these additional technologies, the proposed platform with increased information capacity will enable the practical use of the DNA-based data storage in the future.

## Material and Methods

### The Data to DNA Sequence encoding

For the first demonstration, a text file(txt) describing a brief introduction and member list of the laboratory to which the corresponding author belongs was encoded to DNA (Fig. S1). For the second demonstration, a thumbnail image of Hunminjeongum Manuscript (Fig. S2) was encoded. The image file was resized to 692 × 574 and the file size was 135,393 bytes. Binary data was extracted from the file and grouped as length of DNA fragment. Reed-Solomon redundancy fragments were added for the second demonstration. After that the address were attached. All digits were transformed to DNA codons as described in the Tables S1–S3. More details of data to DNA encoding are described in the Supplementary Note.

### DNA sample preparation and quantification

Oligonucleotides for the first demo were purchased from the Macrogen (Seoul, South Korea). Oligonucleotides of each tube of 100 uM concentration were pooled as one tube and diluted for intended concentration. For the microarray-derived DNA oligopool synthesis, we used B3 Synthesizer DNA microarray synthesizer (Customarray Inc. USA). We synthesized 12 k microarray following standard protocol provided (Customarray Inc. USA). qPCR was utilized for quantification of synthesized DNA oligonucleotide pool. Samples were analysed by qPCR (FAST 7500, Applied Biosystems) using a KAPA SYBR® FAST qPCR Master Mix (2X) Kit. Sample mix of 10 µL master mix, 7 µL of PCR grade water, 1 µL of a 10 µM primer stock of forward and reverse each, 1 µL oligo pool solution was used. We followed standard thermal protocol from the manual. Relative sample quantification was accomplished by interpolation from a standard curve, generated from DNA samples of known concentration. The synthesized DNA library consisted of 1974204 molecules per microliter (438 molecules per fragment). Reported values are averaged from the three replicates (standard deviation: 81969). We used 1ul sample of pooled oligonucleotide synthesized. More details such as primer sequence for PCR are described in the Supplementary Note.

### Amplification and sequencing of DNA

Samples were amplified using qPCR (FAST 7500, Applied Biosystems) and KAPA HiFi Library Amplification Kit. Sample mix of 10 µL master mix, 6 µL of PCR grade water, 1 µL of a 10 µM primer stock of Forward and Reverse each, 1 µL oligo pool solution, 20X SYBR Green was used. We followed standard thermal protocol from the manual. We checked the amplification plot using the qPCR. As soon as the plot reached the saturation, we stopped the machine and purified the sampling using PCR purification kit (Qiagen). We sequenced the amplified oligo pool using on a Miniseq using a 300 cycle pair-end read protocol.

### DNA to data decoding

Pair-end reads of the raw NGS file (Fastq format) were stitched using the PEAR. After that the NGS reads with the appropriate lengths were filtered and duplicated reads were removed. Duplicated reads were removed and representing sequence (include degenerate base) was figured. From the representing sequence, the DNA codon was transformed to digit, by following Supplementary Tables S1–S3. Error correction using Reed-Solomon code was performed for the second demonstration. More details of DNA to data decoding are described in the Supplementary Note.

### Monte Carlo simulation

Data was encoded after random data generation corresponding to one fragment. After that, the read number of fragments was randomly determined following uneven representation of fragments (Fig. S7). Sequencing results for the determined number of reads was generated. In the sequencing results, the base corresponding to the degenerate base were generated randomly corresponding to the binomial distribution (Fig. S6, Supplementary Note), and the mutual probability is the same. Also, error base was generated and p = 2%. If the GC contents are less than 40% or more than 60%, the read was discarded and was generated again. This reflects the low yield of PCR amplification according to GC contents in wet lab experiments. Decoding process was followed. In case of the extended base set (3: 7 or 7: 3), the decision was proceeded by comparing the ratio between the two bases. The whole process was repeated to decoding several tens of gigabytes. For decoding the 100 MB, which is described in the main text, random data of 100 MB was generated at once and decoded. Then the error was corrected. The process was repeated 10 times. For the simulation using 6 encoding characters, the fragment encoded in the experiment was used as an input, and the uneven distribution (Fig. S7) obtained in the experiment was used.

## Supplementary information

Supplementary Information

## Data Availability

The datasets used and/or analyzed during the current study are available from the corresponding author on reasonable request.
