# Supplementary material for: High information capacity DNA-based data storage with augmented encoding characters using degenerate bases
Source: Sci Rep. 2019 Apr 29;9:6582. doi: 10.1038/s41598-019-43105-w (PMC6488701; doi:10.1038/s41598-019-43105-w)
Supplement: Supplementary file 1 — Supplementary Information [file 41598_2019_43105_MOESM1_ESM.docx]

Supplementary Information for

**High information capacity DNA-based data storage with augmented encoding characters using degenerate bases**

Yeongjae Choi, Taehoon Ryu, Amos C. Lee, Hansol Choi, Hansaem Lee, Jaejun Park, Suk-Heung Song, Seojoo Kim, Hyeli Kim, Wook Park and Sunghoon Kwon

Correspondence should be addressed to

S.K. (skwon@snu.ac.kr) or to W.P. (parkwook@khu.ac.kr).

**This file includes:**

Supplementary Notes

Supplementary Figures S1 to S6

Supplementary Tables S1 to S4

Definition of information capacity

The parameter, information capacity, is defined as the ‘Bytes per bases^1^‘, ‘The input information in bits divided by the number of synthesized DNA nucleotides, bits per nucleotide^2^ ’ or ‘Bits per base^3^’ in previous reports. Even though the unit for the term is slightly different between literature, the parameter is used to determine how much data can be encoded in the DNA sequence, not physical molecules, before synthesis of the DNA. Therefore, it is a value that can intuitively convey the performance of a data to DNA encoding algorithm. Moreover, current DNA synthesis methods synthesize more than billions of molecules per sequence, rather than as a single molecule, the parameter is directly related to the amount of use of the synthesizer and is related to the cost as written in this paper.

The value that reflects the number of physical molecule used is described as the “physical density” (ratio of the number of bytes encoded to the weight of the DNA library, Pbyte/g or Pbyte/mm^3^) in both previous researches and our manuscript. The additional use of the parameter “physical density” is necessary to use multiple molecule copies when storing information (single molecule-based DNA-based data storage is not possible yet), due to errors or dropouts of DNA molecules that can occur during DNA synthesis, amplification and sequencing. For example, Erlich *and* Zielinski^2^ experimentally showed that each designed oligonucleotide should be represented by at least ~1300 molecule copy on average to recover the data.­­

DNA synthesis

For the large-scale DNA synthesis, most-used synthesis method is the column-based, such as the Mermade platform from Bioautomation (<https://bioautomation.com>). Using the platform, only the increment of the number of the nozzle, which releases phosphoramidites on the column while synthesis, would be needed to synthesize degenerate base and no additional synthesis cost is not needed when the platform is set. The oligo provider, such as the one we used (Macrogen, https://www.macrogen.com) usually has the setup of the machine and therefore additional cost is not needed.

For the array synthesis, which used for synthesizing pooled oligonucleotide library, there are several methods used such as semiconductor-based (Customarray, http://www.customarrayinc.com), photolithography-based (Nimblegen), or inkjet-based (Agilent(https://www.genomics.agilent.com/article.jsp?pageId=2011) and Twist Bioscience (<https://twistbioscience.com)>). Among these technology, the inkjet-based array synthesis would be applied to the introduced platform, with the increment of the nozzle. However, for other methods, use of the degenerate base while synthesis could result the increment of the steps of the cycles while synthesis and the cost decrement would not be expected.

Data to DNA encoding: encoded files

For the first demo, we encoded a text file(txt) describing a brief introduction and member list of the laboratory to which the corresponding author belongs (Supplementary Fig. 1).

For the second demo, we encoded a thumbnail image of Hunminjeongum Manuscript (or Hunminjeongum Haerye, Supplementary Fig. 2), which is the UNESCO memory of the world registered documented heritage submitted by Republic of Korea in 1997(http://www.unesco.org/new/en/communication-and-information/memory-of-the-world/register/full-list-of-registered-heritage/registered-heritage-page-8/the-hunmin-chongum-manuscript/). Please see the link for further detail. Image file was resized to 692 × 574 and the file size was 135,393 bytes.

Data to DNA encoding

We encode the data as DNA by using the process introduced by Grass et al.^4^*.*

For the first demo:

1. Binary data was extracted from the file and the total data was grouped as 19bits. The 19bits were transformed into 2 DNA codons of Supplementary Table 1.

2. DNA sequence was fragmented as 42nt.

3. Addresses were attached. Address digits were transformed to DNA codons as described in Supplementary Table 2.

For the second demo:

1. Binary data was extracted from the image and the total data was fragmented after each 7bit was grouped. Fragmented length was 37 X 7 bit.

2. Reed-Solomon redundancy fragments were added.

3. Addresses were attached

4. Digits were transformed to DNA codons as described in Supplementary Table 2, 3

The encoded information is divided into fragments of 111 nt, and an address composed of non-degenerative nucleotides of a length of 9 nt is assigned thereto. Each fragment is supplemented with an adapter for amplification and sequencing, and the entire fragment is 160 nt in length. We also add Reed-Solomon based redundancy block in order to cope with errors that may occur during DNA synthesis, storage, and sequencing (Supplementary Fig. 4). Depending on the ratio of the size of the Reed-Solomon block to the information block size, the error correction capability and the data density are traded-off. We designed 9 redundancy fragments in 118 information fragments to correct 3.5% false information or 7% missing information in maximum.

Data to DNA encoding: Reed-Solomon error correction

We only use the outer error correction code (Supplementary Fig. 4). Reed Solomon codes with parameters (n-total length, k-message length) follow the relation:

2e + f ≤ n-k, e: number of errors f: number of erasures

For all Reed-Solomon error correction, we use base 128 number system (or see each 7bit as digit) to match with the codon with degenerate bases.

4183 fragments in total were generated from the data fragmentizing and address attaching process. Fragments were divided into 35 blocks of 118 fragments and one block of 53 fragments. We added 9 redundancy fragments in the blocks of 118 fragments. Also, 5 redundancy fragments were added in block of 53 fragments. Finally, 4503 fragments in total were generated.

Data to DNA encoding: Address attaching

Each fragment was matched to addresses consisting of three digits of 48 base number system, which is matched with codon without degenerative bases (Supplementary Table 2). For the second demo, the system can encode about 110,000 fragments, which is about 3 MB of encoding capability. To encode data in few gigabytes, additional space of 6nt (or 2 more digit) is needed. For the second demo, address sequence was reversed, to avoid error in cluster identification during Illumina sequencing due to the homo polymer sequence.

Data to DNA encoding: Codon table

1. We have created a codon table with a degenerative sequence added. The sequence of the last position of the 3 nt codons was not the same as that of the front sequence in order to avoid homopolymer of 4 base pairs or more. The W and S sequences correspond to both A, T and C, G respectively. This gives us total 750 codons (Supplementary Table 1) for the first demo and 132 codons for the second demo (Supplementary Table 3). Data digits generated in previous step were transformed using the table.
2. Each fragment was matched to addresses consisting of three 48 codons without degenerative (Supplementary Table 2). Address digits generated in the previous step were transformed using the table.

Data to DNA encoding: Adaptor sequence

The adapter sequence was made by trimming the sequencing primer of the Illumina system.

Forward adaptor: ACACGACGCTCTTCCGATCT

Reverse adaptor: AGATCGGAAGAGCACACGTC

Synthesized oligonucleotide pool quantification

qPCR was utilized for quantification of synthesized DNA oligonucleotide pool. Samples were analyzed by qPCR (FAST 7500, Applied Biosystems) using a KAPA SYBR® FAST qPCR Master Mix (2X) Kit. Sample mix of 10 µL master mix, 7 µL of PCR grade water, 1 µL of a 10 µM primer stock of forward and reverse each, 1 µL oligo pool solution was used. We followed standard thermal protocol from the manual. Sequences of the forward and reverse primer are:

Multiplexing Read 1 Sequencing Primer (Forward)

5' ACACTCTTTCCCTACACGACGCTCTTCCGATCT

Multiplexing Read 2 Sequencing Primer (Reverse)

5' GTGACTGGAGTTCAGACGTGTGCTCTTCCGATCT

Relative sample quantification was accomplished by interpolation from a standard curve, generated from DNA samples of known concentration. The synthesized DNA library consisted of 1974204 molecules per microliter (438 molecules per fragment). Reported values are averaged from the three replicates (standard deviation: 81969). We used 1ul sample of pooled oligonucleotide synthesized.

DNA to Data decoding: Overview

1. Pair-end reads of the raw Fastq file were stitched using the PEAR.
2. NGS reads with the appropriate lengths were filtered.
3. Duplicated reads were removed.
4. Reads were categorized by addresses.
5. Representing sequence (include degenerate base) was figured.
6. The DNA codon was transformed to digit
7. Error correction using Reed-Solomon code was performed

DNA to Data decoding: length filtering, duplication removing, categorizing reads by address

1. Pair-end reads of the raw Fastq file were stitched using the PEAR. assembly length value (-m, -n) were specified as 47 and 43 for the first demo, since the designed length of the DNA is shorter than its default value.
2. For the first demo, reads were filtered based on the quality score in address position (Q-score over 40).
3. Only NGS reads with the appropriate length (45 for the first demo, 120nt for the second) were used for decoding.
4. Reads were categorized by address. The address sequences were decoded using Table S2.
5. Duplicated reads were removed

Supplementary Table 4 describes the number of reads obtained in step 1, 3, 5 in Raw NGS data.

To see the assignment error between the address, we checked the false assignment rate from the data of first demo. After we categorized the read by address, we checked the whether the read is appropriately categorized by comparing the original design. In this step, if the read is more than 50% different from the design (in the case of degenerate base, all the combination in the base are considered when compared), we considered this as the false assign due to the crosstalk. The average rate of false assignment is 0.18%, and the maximum value was 0.93%.

DNA to Data decoding: Transform the DNA codon to digit

1. DNA sequences were divided into 3nt and transformed to the 7bit digit, by following the codon listed in Supplementary Table 1 or 3.

If there was no codon matched to the digit, it was categorized as ‘erased’ for further error correction in next step.

DNA to Data decoding: Error correction using Reed-Solomon code

We corrected error by process introduced from Grass et al^4^.

1. For the 2.0 bits/character model, outer Reed-Solomon code was used for error correction (Supplementary Fig. 4).

Binomial distribution confirmation for Supplementary Fig. 6

1. Fragments with more than 50 read calls were selected. After that, 50 reads were randomly sampled per fragment.
2. If we draw a histogram of the elements that form the degenerate base (Supplementary Fig. 6, blue line), the histogram could be fit into the binomial distribution (red line), which follows the equation:

$$P\left( x \right)=p^{x}{(1-p)}^{n-x}\binom{n}{x}$$

in which, n is 50. Also, from the fitted distribution, we could extract value p, which was used from the simulation.

Calculation of physical density

Physical density of the DNA-based data storage is ratio of the number of bytes encoded to the weight of the DNA library. The calculation method is,

$$Physical density({Pbyte}/g)= \frac{Quantity of stored data}{Weight of DNA molecule used(g/{single}molecule)*number of molecules used for storage}$$

From this, we calculate the physical density of our result as;

1. Molecular weight of DNA molecule used: (length of oligonucleotide) * 303.7 + 79 (g/mol) :

- For experiment used 15 encoding characters: 85 nucleotides
- For experiment used 6 encoding characters: 160 nucleotides
- Gram per mole was converted to gram per single molecule

1. Number of molecules used for storage:

- For experiment used 15 encoding characters: 40 (library number) * 800 (molecular copy)
- For experiment used 6 encoding characters: 4503 (library number) * 438 (molecular copy)

1. Quantity of stored data:

- For experiment used 15 encoding characters: 854 bytes
- For experiment used 15 encoding characters:135.4 Kbytes

Cost projection and calculation of information capacity

1. If 15 encoding characters are used for data storage, 750 3-base-codons are generated, by using the method described above. Also, for the case using 26 encoding characters, 1932 codons could be generated. Here, the information capacity is obtained as:

$$\frac{\log_{2} \left( Number of codons \right)}{3} bits/character$$

2. The capacity should be multiplied by the reduction factor of the information capacity due to the address used in the design. The reduction factor is:

$$\frac{148 (length excluding addresses)}{160 (length including addresses)}$$

3. The resulting value is multiplied by factor of 1 / 1.1 to give the error correction of 10%. This gives us the information capacity described in the text.
4. To reflect the length of the adapter for PCR amplification, the resulting value should be multiplied by:

$$\frac{160 (length used for information storage)}{200 (full length when adapter is used)}$$

5. The number of nucleotides storing 1MB is calculated by:

$$\frac{1MB}{Information capacity reflecting adapter}$$

The information capacity of other studies follows the values summarized in previous studies.

The estimated number of nucleotides is multiplied by the synthesis cost per nucleotides to get the price described in the manuscript. Also, NGS cost was estimated using the estimated number of nucleotides and the NGS coverage used in previous studies.


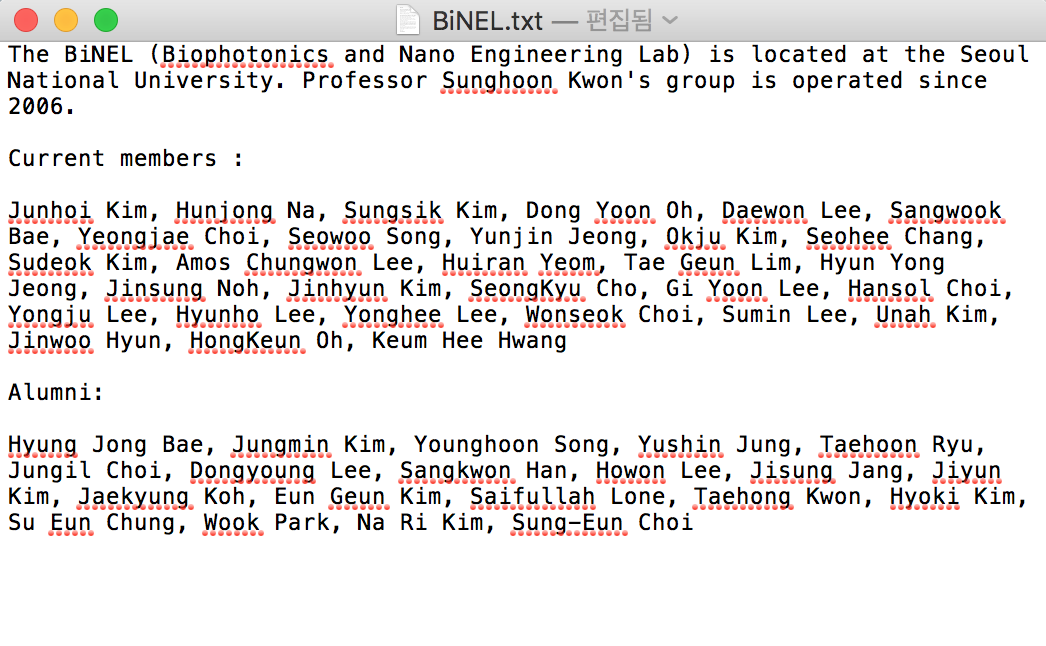


Supplementary Figure S1

The text file used for encoding in the first demonstration. The content of the text file is a member list of the research group.


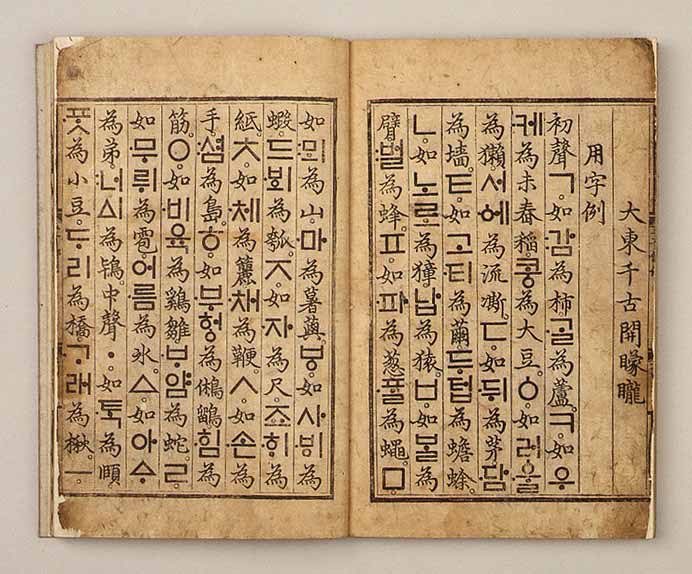


Supplementary Figure S2

The thumbnail image of Hunminjeongum Manuscript (or Hunminjeongum Haerye), which is the UNESCO memory of the world registered documented heritage submitted by Republic of Korea in 1997. (http://heritage.go.kr/heri/cul/culSelectDetail.do?region=1&searchCondition=&searchCondition2=&s_kdcd=11&s_ctcd=00&ccbaKdcd=11&ccbaAsno=00700000&ccbaCtcd=11&ccbaCpno=1111100700000&ccbaCndt=&stCcbaAsno=70&endCcbaAsno=70&stCcbaAsdt=&endCcbaAsdt=&ccbaPcd1=99&culPageNo=1&chGubun=&header=view&returnUrl=%2Fheri%2Fcul%2FculSelectViewList.do&sCond=)

This image was originally posted by the Cultural Heritage Administration of the Republic of Korea under the Korea Open Government License (https://www.mcst.go.kr/kor/s_open/kogl/koglType.jsp?pTab=05) type 1.


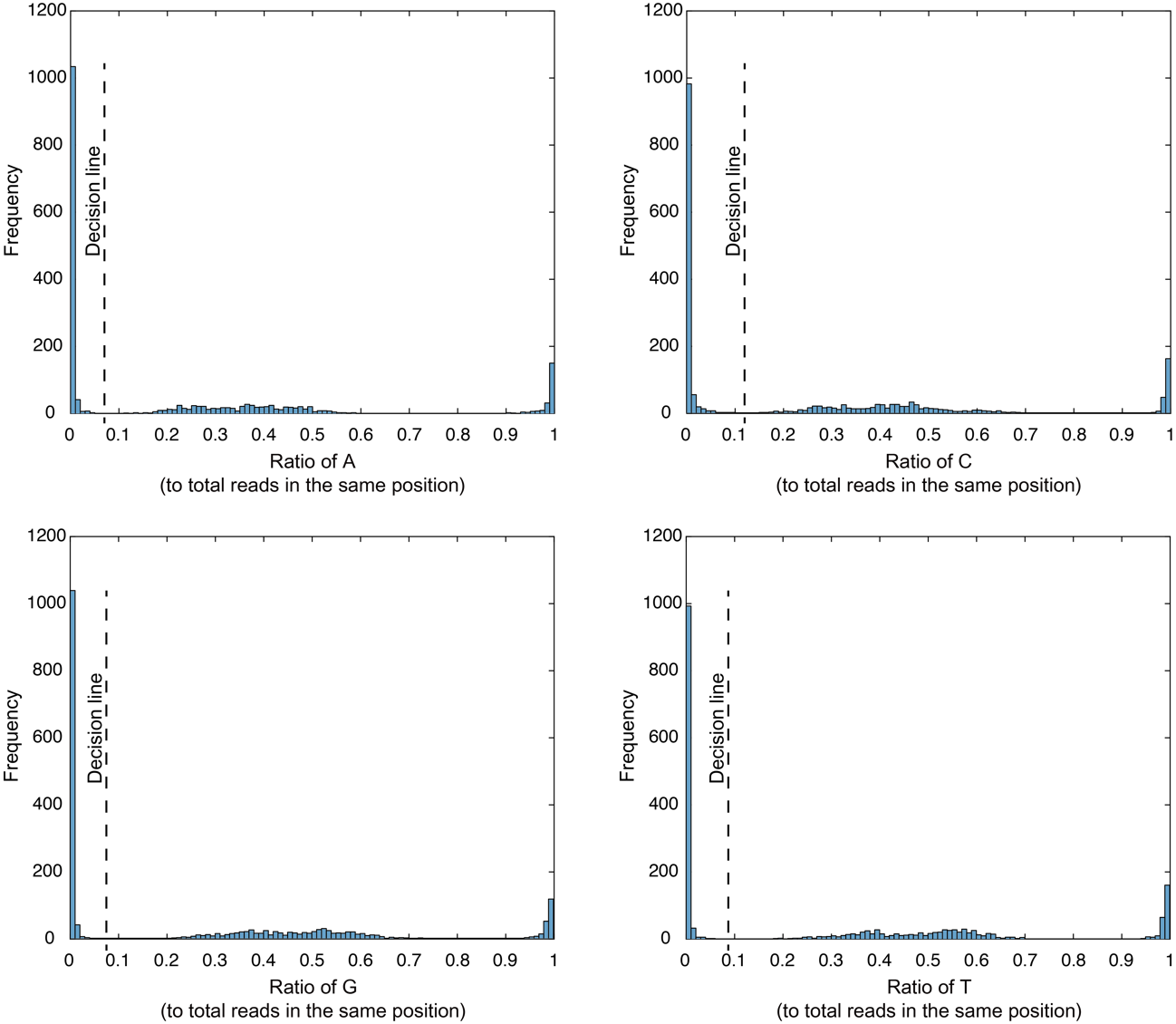


Supplementary Figure S3

The histogram of the ratio of base in a position in the sequence.


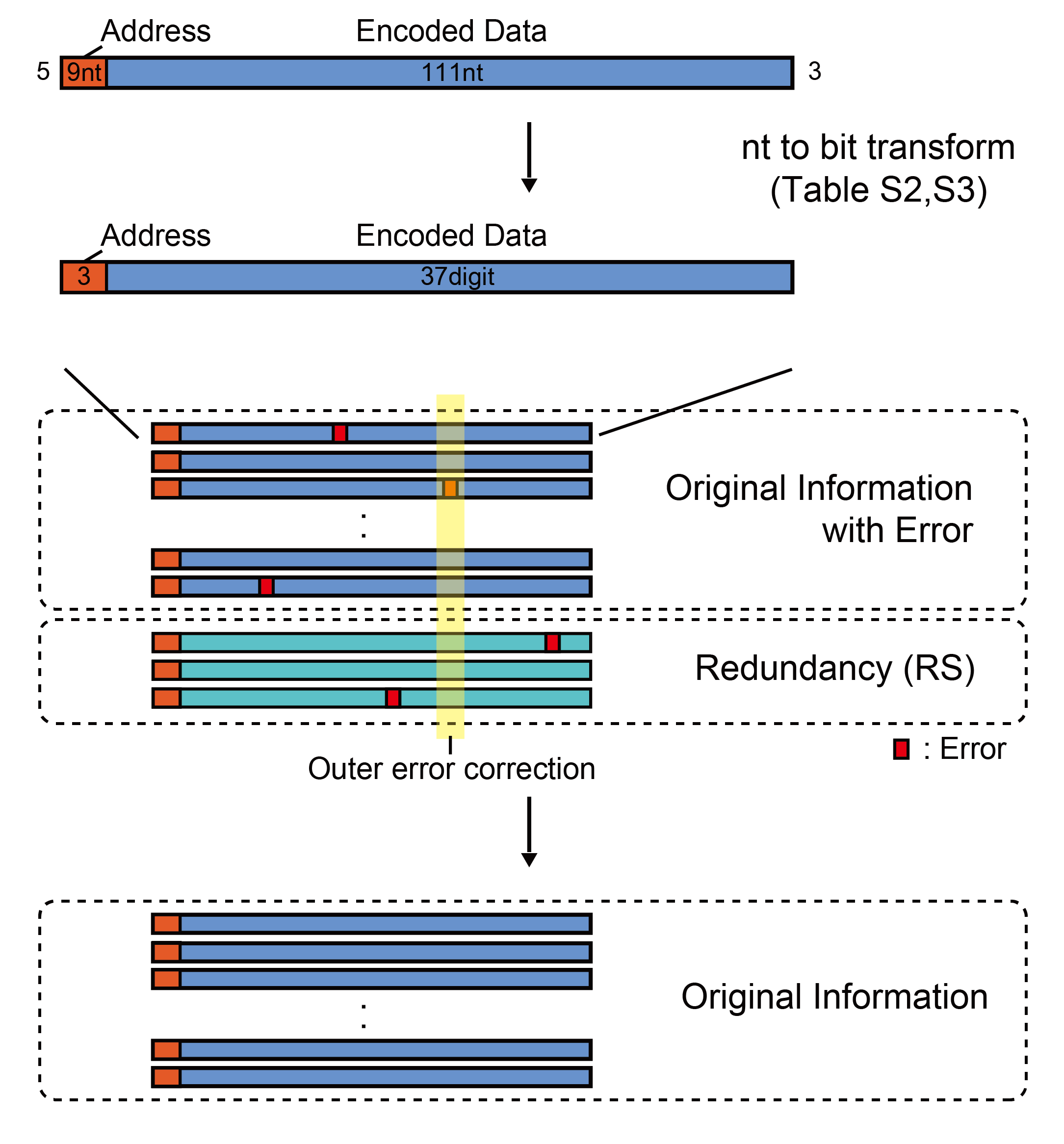


Supplementary Figure S4

Structure of the data fragment (without adaptor) and error correcting scheme for the 2.0 bits/character model.


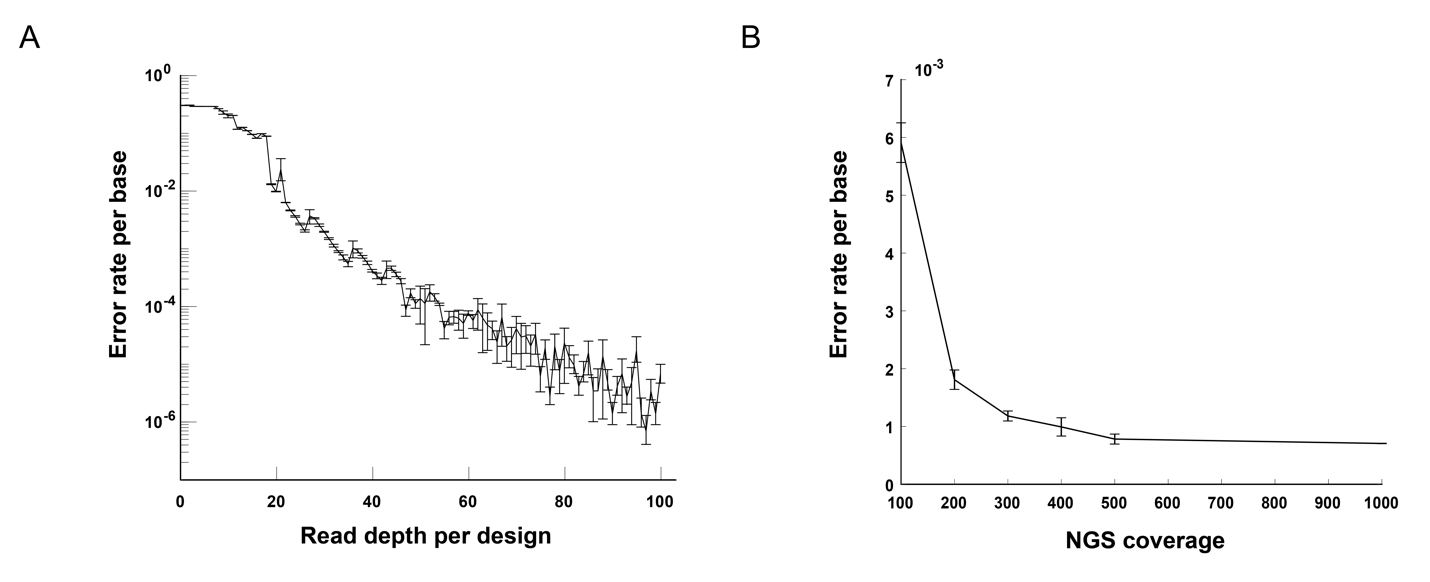


Supplementary Figure S5

A. The error rate per sequenced base-pairs according to depth of different designs, from which the reads were randomly and uniformly sampled (See supplementary information for details). The standard deviations were obtained by repeating the random sampling 10 times. Bars represent one standard deviation from the mean efficiency.

B. The error rate of synthesized base pairs in fragments of specific average coverage over the total fragments. The standard deviations were obtained by repeating the random sampling 10 times. Bars represent one standard deviation from the mean efficiency.


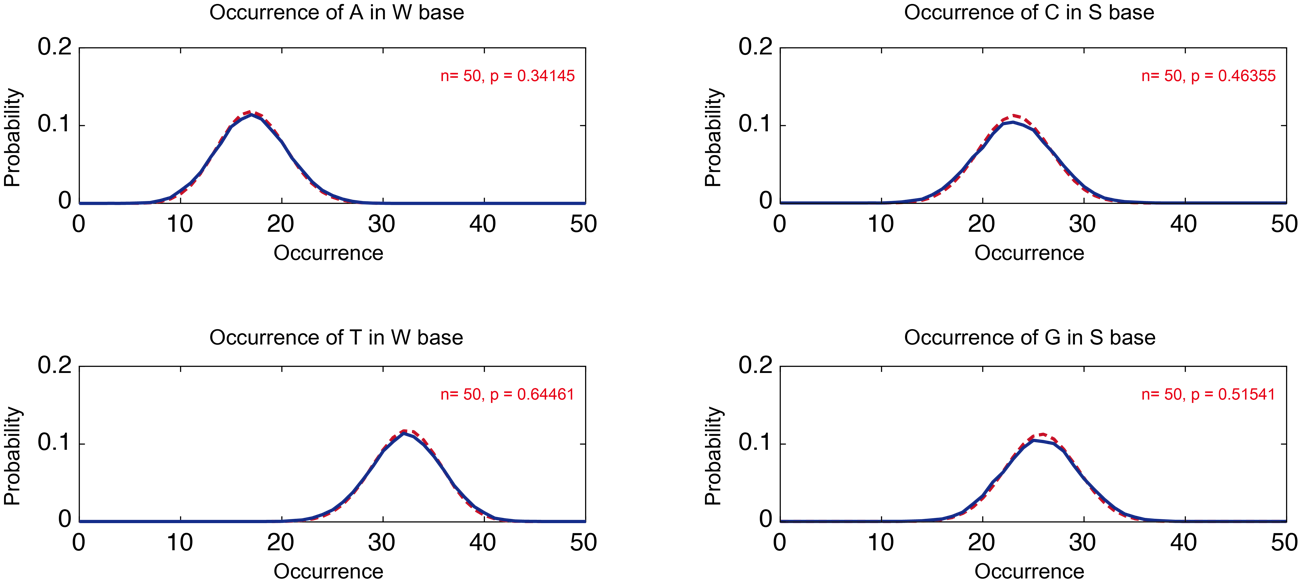


Supplementary Figure S6

Occurrence of original nucleotide that comprises a degenerate base. Blue: histogram, Red: Fitted binomial graph. This is experimental data from the second demonstration where we used W and S.


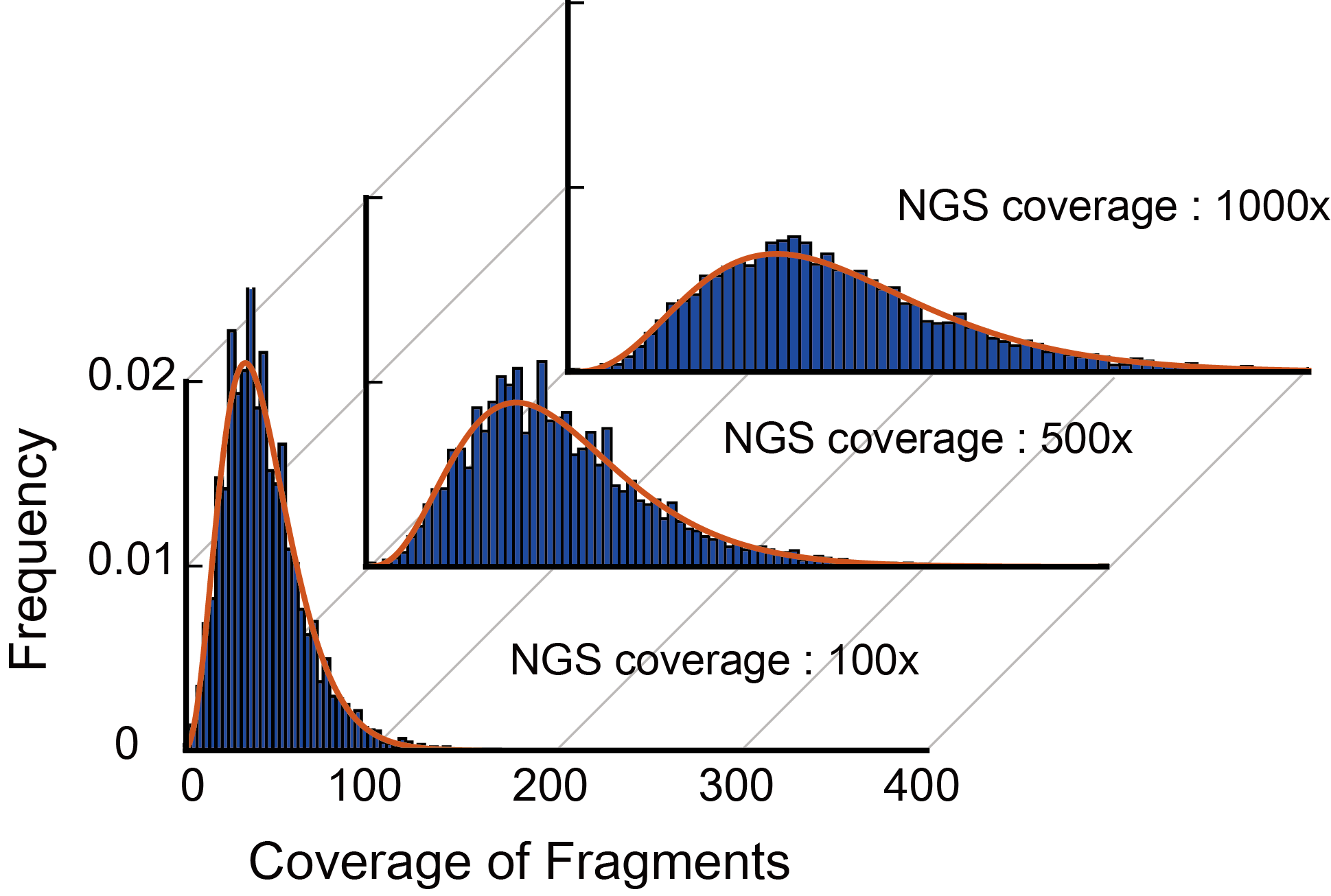


Supplementary Figure S7

Profile of uneven representation of fragments due to the PCR bias, which was obtained from the second experiment using six encoding alphabets. Red, negative binomial fit following previous reports

Supplementary Table S1

Codon table with degenerative sequence used for data encoding.

| ACA | ZTA | OYA | USA | DIA | BXA |
| --- | --- | --- | --- | --- | --- |
| CCA | XTA | PYA | ISA | NIA | VXA |
| TCA | AGA | ZYA | OSA | UIA | DXA |
| GCA | CGA | XYA | PSA | IIA | NXA |
| RCA | TGA | AKA | ZSA | OIA | UXA |
| YCA | GGA | CKA | XSA | PIA | IXA |
| MCA | RGA | TKA | ABA | ZIA | OXA |
| KCA | YGA | GKA | CBA | XIA | PXA |
| WCA | MGA | RKA | TBA | APA | ZXA |
| SCA | KGA | YKA | GBA | CPA | XXA |
| HCA | WGA | MKA | RBA | TPA | AAC |
| BCA | SGA | KKA | YBA | GPA | CAC |
| VCA | HGA | WKA | MBA | RPA | TAC |
| DCA | BGA | SKA | KBA | YPA | GAC |
| NCA | VGA | HKA | WBA | MPA | RAC |
| UCA | DGA | BKA | SBA | KPA | YAC |
| ICA | NGA | VKA | HBA | WPA | MAC |
| OCA | UGA | DKA | BBA | SPA | KAC |
| PCA | IGA | NKA | VBA | HPA | WAC |
| ZCA | OGA | UKA | DBA | BPA | SAC |
| XCA | PGA | IKA | NBA | VPA | HAC |
| ATA | ZGA | OKA | UBA | DPA | BAC |
| CTA | XGA | PKA | IBA | NPA | VAC |
| TTA | AYA | ZKA | OBA | UPA | DAC |
| GTA | CYA | XKA | PBA | IPA | NAC |
| RTA | TYA | ASA | ZBA | OPA | UAC |
| YTA | GYA | CSA | XBA | PPA | IAC |
| MTA | RYA | TSA | AIA | ZPA | OAC |
| KTA | YYA | GSA | CIA | XPA | PAC |
| WTA | MYA | RSA | TIA | AXA | ZAC |
| STA | KYA | YSA | GIA | CXA | XAC |
| HTA | WYA | MSA | RIA | TXA | ATC |
| BTA | SYA | KSA | YIA | GXA | CTC |
| VTA | HYA | WSA | MIA | RXA | TTC |
| DTA | BYA | SSA | KIA | YXA | GTC |
| NTA | VYA | HSA | WIA | MXA | RTC |
| UTA | DYA | BSA | SIA | KXA | YTC |
| ITA | NYA | VSA | HIA | WXA | MTC |
| OTA | UYA | DSA | BIA | SXA | KTC |
| PTA | IYA | NSA | VIA | HXA | WTC |

| STC | KRC | YWC | GUC | CZC | XAT |
| --- | --- | --- | --- | --- | --- |
| HTC | WRC | MWC | RUC | TZC | ACT |
| BTC | SRC | KWC | YUC | GZC | CCT |
| VTC | HRC | WWC | MUC | RZC | TCT |
| DTC | BRC | SWC | KUC | YZC | GCT |
| NTC | VRC | HWC | WUC | MZC | RCT |
| UTC | DRC | BWC | SUC | KZC | YCT |
| ITC | NRC | VWC | HUC | WZC | MCT |
| OTC | URC | DWC | BUC | SZC | KCT |
| PTC | IRC | NWC | VUC | HZC | WCT |
| ZTC | ORC | UWC | DUC | BZC | SCT |
| XTC | PRC | IWC | NUC | VZC | HCT |
| AGC | ZRC | OWC | UUC | DZC | BCT |
| CGC | XRC | PWC | IUC | NZC | VCT |
| TGC | AKC | ZWC | OUC | UZC | DCT |
| GGC | CKC | XWC | PUC | IZC | NCT |
| RGC | TKC | ADC | ZUC | OZC | UCT |
| YGC | GKC | CDC | XUC | PZC | ICT |
| MGC | RKC | TDC | APC | ZZC | OCT |
| KGC | YKC | GDC | CPC | XZC | PCT |
| WGC | MKC | RDC | TPC | AAT | ZCT |
| SGC | KKC | YDC | GPC | CAT | XCT |
| HGC | WKC | MDC | RPC | TAT | AGT |
| BGC | SKC | KDC | YPC | GAT | CGT |
| VGC | HKC | WDC | MPC | RAT | TGT |
| DGC | BKC | SDC | KPC | YAT | GGT |
| NGC | VKC | HDC | WPC | MAT | RGT |
| UGC | DKC | BDC | SPC | KAT | YGT |
| IGC | NKC | VDC | HPC | WAT | MGT |
| OGC | UKC | DDC | BPC | SAT | KGT |
| PGC | IKC | NDC | VPC | HAT | WGT |
| ZGC | OKC | UDC | DPC | BAT | SGT |
| XGC | PKC | IDC | NPC | VAT | HGT |
| ARC | ZKC | ODC | UPC | DAT | BGT |
| CRC | XKC | PDC | IPC | NAT | VGT |
| TRC | AWC | ZDC | OPC | UAT | DGT |
| GRC | CWC | XDC | PPC | IAT | NGT |
| RRC | TWC | AUC | ZPC | OAT | UGT |
| YRC | GWC | CUC | XPC | PAT | IGT |
| MRC | RWC | TUC | AZC | ZAT | OGT |

| PGT | IMT | NVT | VOT | HAG | WTG |
| --- | --- | --- | --- | --- | --- |
| ZGT | OMT | UVT | DOT | BAG | STG |
| XGT | PMT | IVT | NOT | VAG | HTG |
| ART | ZMT | OVT | UOT | DAG | BTG |
| CRT | XMT | PVT | IOT | NAG | VTG |
| TRT | AST | ZVT | OOT | UAG | DTG |
| GRT | CST | XVT | POT | IAG | NTG |
| RRT | TST | AUT | ZOT | OAG | UTG |
| YRT | GST | CUT | XOT | PAG | ITG |
| MRT | RST | TUT | AXT | ZAG | OTG |
| KRT | YST | GUT | CXT | XAG | PTG |
| WRT | MST | RUT | TXT | ACG | ZTG |
| SRT | KST | YUT | GXT | CCG | XTG |
| HRT | WST | MUT | RXT | TCG | AYG |
| BRT | SST | KUT | YXT | GCG | CYG |
| VRT | HST | WUT | MXT | RCG | TYG |
| DRT | BST | SUT | KXT | YCG | GYG |
| NRT | VST | HUT | WXT | MCG | RYG |
| URT | DST | BUT | SXT | KCG | YYG |
| IRT | NST | VUT | HXT | WCG | MYG |
| ORT | UST | DUT | BXT | SCG | KYG |
| PRT | IST | NUT | VXT | HCG | WYG |
| ZRT | OST | UUT | DXT | BCG | SYG |
| XRT | PST | IUT | NXT | VCG | HYG |
| AMT | ZST | OUT | UXT | DCG | BYG |
| CMT | XST | PUT | IXT | NCG | VYG |
| TMT | AVT | ZUT | OXT | UCG | DYG |
| GMT | CVT | XUT | PXT | ICG | NYG |
| RMT | TVT | AOT | ZXT | OCG | UYG |
| YMT | GVT | COT | XXT | PCG | IYG |
| MMT | RVT | TOT | AAG | ZCG | OYG |
| KMT | YVT | GOT | CAG | XCG | PYG |
| WMT | MVT | ROT | TAG | ATG | ZYG |
| SMT | KVT | YOT | GAG | CTG | XYG |
| HMT | WVT | MOT | RAG | TTG | AMG |
| BMT | SVT | KOT | YAG | GTG | CMG |
| VMT | HVT | WOT | MAG | RTG | TMG |
| DMT | BVT | SOT | KAG | YTG | GMG |
| NMT | VVT | HOT | WAG | MTG | RMG |
| UMT | DVT | BOT | SAG | KTG | YMG |

| MMG | RHG | TOG | ACR | ZTR | OIR |
| --- | --- | --- | --- | --- | --- |
| KMG | YHG | GOG | CCR | XTR | PIR |
| WMG | MHG | ROG | TCR | AYR | ZIR |
| SMG | KHG | YOG | GCR | CYR | XIR |
| HMG | WHG | MOG | RCR | TYR | AAY |
| BMG | SHG | KOG | YCR | GYR | CAY |
| VMG | HHG | WOG | MCR | RYR | TAY |
| DMG | BHG | SOG | KCR | YYR | GAY |
| NMG | VHG | HOG | WCR | MYR | RAY |
| UMG | DHG | BOG | SCR | KYR | YAY |
| IMG | NHG | VOG | HCR | WYR | MAY |
| OMG | UHG | DOG | BCR | SYR | KAY |
| PMG | IHG | NOG | VCR | HYR | WAY |
| ZMG | OHG | UOG | DCR | BYR | SAY |
| XMG | PHG | IOG | NCR | VYR | HAY |
| AWG | ZHG | OOG | UCR | DYR | BAY |
| CWG | XHG | POG | ICR | NYR | VAY |
| TWG | AIG | ZOG | OCR | UYR | DAY |
| GWG | CIG | XOG | PCR | IYR | NAY |
| RWG | TIG | AZG | ZCR | OYR | UAY |
| YWG | GIG | CZG | XCR | PYR | IAY |
| MWG | RIG | TZG | ATR | ZYR | OAY |
| KWG | YIG | GZG | CTR | XYR | PAY |
| WWG | MIG | RZG | TTR | AIR | ZAY |
| SWG | KIG | YZG | GTR | CIR | XAY |
| HWG | WIG | MZG | RTR | TIR | AGY |
| BWG | SIG | KZG | YTR | GIR | CGY |
| VWG | HIG | WZG | MTR | RIR | TGY |
| DWG | BIG | SZG | KTR | YIR | GGY |
| NWG | VIG | HZG | WTR | MIR | RGY |
| UWG | DIG | BZG | STR | KIR | YGY |
| IWG | NIG | VZG | HTR | WIR | MGY |
| OWG | UIG | DZG | BTR | SIR | KGY |
| PWG | IIG | NZG | VTR | HIR | WGY |
| ZWG | OIG | UZG | DTR | BIR | SGY |
| XWG | PIG | IZG | NTR | VIR | HGY |
| AHG | ZIG | OZG | UTR | DIR | BGY |
| CHG | XIG | PZG | ITR | NIR | VGY |
| THG | AOG | ZZG | OTR | UIR | DGY |
| GHG | COG | XZG | PTR | IIR | NGY |

| UGY | DUY | BGM | SPM | KCK | YOK |
| --- | --- | --- | --- | --- | --- |
| IGY | NUY | VGM | HPM | WCK | MOK |
| OGY | UUY | DGM | BPM | SCK | KOK |
| PGY | IUY | NGM | VPM | HCK | WOK |
| ZGY | OUY | UGM | DPM | BCK | SOK |
| XGY | PUY | IGM | NPM | VCK | HOK |
| ARY | ZUY | OGM | UPM | DCK | BOK |
| CRY | XUY | PGM | IPM | NCK | VOK |
| TRY | ATM | ZGM | OPM | UCK | DOK |
| GRY | CTM | XGM | PPM | ICK | NOK |
| RRY | TTM | AKM | ZPM | OCK | UOK |
| YRY | GTM | CKM | XPM | PCK | IOK |
| MRY | RTM | TKM | AAK | ZCK | OOK |
| KRY | YTM | GKM | CAK | XCK | POK |
| WRY | MTM | RKM | TAK | AMK | ZOK |
| SRY | KTM | YKM | GAK | CMK | XOK |
| HRY | WTM | MKM | RAK | TMK | ACW |
| BRY | STM | KKM | YAK | GMK | CCW |
| VRY | HTM | WKM | MAK | RMK | TCW |
| DRY | BTM | SKM | KAK | YMK | GCW |
| NRY | VTM | HKM | WAK | MMK | RCW |
| URY | DTM | BKM | SAK | KMK | YCW |
| IRY | NTM | VKM | HAK | WMK | MCW |
| ORY | UTM | DKM | BAK | SMK | KCW |
| PRY | ITM | NKM | VAK | HMK | WCW |
| ZRY | OTM | UKM | DAK | BMK | SCW |
| XRY | PTM | IKM | NAK | VMK | HCW |
| AUY | ZTM | OKM | UAK | DMK | BCW |
| CUY | XTM | PKM | IAK | NMK | VCW |
| TUY | AGM | ZKM | OAK | UMK | DCW |
| GUY | CGM | XKM | PAK | IMK | NCW |
| RUY | TGM | APM | ZAK | OMK | UCW |
| YUY | GGM | CPM | XAK | PMK | ICW |
| MUY | RGM | TPM | ACK | ZMK | OCW |
| KUY | YGM | GPM | CCK | XMK | PCW |
| WUY | MGM | RPM | TCK | AOK | ZCW |
| SUY | KGM | YPM | GCK | COK | XCW |
| HUY | WGM | MPM | RCK | TOK | AGW |
| BUY | SGM | KPM | YCK | GOK | CGW |
| VUY | HGM | WPM | MCK | ROK | TGW |

| GGW | CXW | XAS | PWS | IGH | NTV |
| --- | --- | --- | --- | --- | --- |
| RGW | TXW | ATS | ZWS | OGH | UTV |
| YGW | GXW | CTS | XWS | PGH | ITV |
| MGW | RXW | TTS | AZS | ZGH | OTV |
| KGW | YXW | GTS | CZS | XGH | PTV |
| WGW | MXW | RTS | TZS | AAB | ZTV |
| SGW | KXW | YTS | GZS | CAB | XTV |
| HGW | WXW | MTS | RZS | TAB | ACD |
| BGW | SXW | KTS | YZS | GAB | CCD |
| VGW | HXW | WTS | MZS | RAB | TCD |
| DGW | BXW | STS | KZS | YAB | GCD |
| NGW | VXW | HTS | WZS | MAB | RCD |
| UGW | DXW | BTS | SZS | KAB | YCD |
| IGW | NXW | VTS | HZS | WAB | MCD |
| OGW | UXW | DTS | BZS | SAB | KCD |
| PGW | IXW | NTS | VZS | HAB | WCD |
| ZGW | OXW | UTS | DZS | BAB | SCD |
| XGW | PXW | ITS | NZS | VAB | HCD |
| ASW | ZXW | OTS | UZS | DAB | BCD |
| CSW | XXW | PTS | IZS | NAB | VCD |
| TSW | AAS | ZTS | OZS | UAB | DCD |
| GSW | CAS | XTS | PZS | IAB | NCD |
| RSW | TAS | AWS | ZZS | OAB | UCD |
| YSW | GAS | CWS | XZS | PAB | ICD |
| MSW | RAS | TWS | AGH | ZAB | OCD |
| KSW | YAS | GWS | CGH | XAB | PCD |
| WSW | MAS | RWS | TGH | ATV | ZCD |
| SSW | KAS | YWS | GGH | CTV | XCD |
| HSW | WAS | MWS | RGH | TTV | ACU |
| BSW | SAS | KWS | YGH | GTV | CCU |
| VSW | HAS | WWS | MGH | RTV | TCU |
| DSW | BAS | SWS | KGH | YTV | GCU |
| NSW | VAS | HWS | WGH | MTV | RCU |
| USW | DAS | BWS | SGH | KTV | YCU |
| ISW | NAS | VWS | HGH | WTV | MCU |
| OSW | UAS | DWS | BGH | STV | KCU |
| PSW | IAS | NWS | VGH | HTV | WCU |
| ZSW | OAS | UWS | DGH | BTV | SCU |
| XSW | PAS | IWS | NGH | VTV | HCU |
| AXW | ZAS | OWS | UGH | DTV | BCU |

| VCU | HYU | WAI | MRI | RTO | TKO |
| --- | --- | --- | --- | --- | --- |
| DCU | BYU | SAI | KRI | YTO | GKO |
| NCU | VYU | HAI | WRI | MTO | RKO |
| UCU | DYU | BAI | SRI | KTO | YKO |
| ICU | NYU | VAI | HRI | WTO | MKO |
| OCU | UYU | DAI | BRI | STO | KKO |
| PCU | IYU | NAI | VRI | HTO | WKO |
| ZCU | OYU | UAI | DRI | BTO | SKO |
| XCU | PYU | IAI | NRI | VTO | HKO |
| ATU | ZYU | OAI | URI | DTO | BKO |
| CTU | XYU | PAI | IRI | NTO | VKO |
| TTU | AIU | ZAI | ORI | UTO | DKO |
| GTU | CIU | XAI | PRI | ITO | NKO |
| RTU | TIU | AGI | ZRI | OTO | UKO |
| YTU | GIU | CGI | XRI | PTO | IKO |
| MTU | RIU | TGI | AUI | ZTO | OKO |
| KTU | YIU | GGI | CUI | XTO | PKO |
| WTU | MIU | RGI | TUI | AGO | ZKO |
| STU | KIU | YGI | GUI | CGO | XKO |
| HTU | WIU | MGI | RUI | TGO | APO |
| BTU | SIU | KGI | YUI | GGO | CPO |
| VTU | HIU | WGI | MUI | RGO | TPO |
| DTU | BIU | SGI | KUI | YGO | GPO |
| NTU | VIU | HGI | WUI | MGO | RPO |
| UTU | DIU | BGI | SUI | KGO | YPO |
| ITU | NIU | VGI | HUI | WGO | MPO |
| OTU | UIU | DGI | BUI | SGO | KPO |
| PTU | IIU | NGI | VUI | HGO | WPO |
| ZTU | OIU | UGI | DUI | BGO | SPO |
| XTU | PIU | IGI | NUI | VGO | HPO |
| AYU | ZIU | OGI | UUI | DGO | BPO |
| CYU | XIU | PGI | IUI | NGO | VPO |
| TYU | AAI | ZGI | OUI | UGO | DPO |
| GYU | CAI | XGI | PUI | IGO | NPO |
| RYU | TAI | ARI | ZUI | OGO | UPO |
| YYU | GAI | CRI | XUI | PGO | IPO |
| MYU | RAI | TRI | ATO | ZGO | OPO |
| KYU | YAI | GRI | CTO | XGO | PPO |
| WYU | MAI | RRI | TTO | AKO | ZPO |
| SYU | KAI | YRI | GTO | CKO | XPO |

| AAP | ZCP | OOP | UGZ | DXZ | BTX |
| --- | --- | --- | --- | --- | --- |
| CAP | XCP | POP | IGZ | NXZ | VTX |
| TAP | AMP | ZOP | OGZ | UXZ | DTX |
| GAP | CMP | XOP | PGZ | IXZ | NTX |
| RAP | TMP | ACZ | ZGZ | OXZ | UTX |
| YAP | GMP | CCZ | XGZ | PXZ | ITX |
| MAP | RMP | TCZ | ASZ | ZXZ | OTX |
| KAP | YMP | GCZ | CSZ | XXZ | PTX |
| WAP | MMP | RCZ | TSZ | AAX | ZTX |
| SAP | KMP | YCZ | GSZ | CAX | XTX |
| HAP | WMP | MCZ | RSZ | TAX | AWX |
| BAP | SMP | KCZ | YSZ | GAX | CWX |
| VAP | HMP | WCZ | MSZ | RAX | TWX |
| DAP | BMP | SCZ | KSZ | YAX | GWX |
| NAP | VMP | HCZ | WSZ | MAX | RWX |
| UAP | DMP | BCZ | SSZ | KAX | YWX |
| IAP | NMP | VCZ | HSZ | WAX | MWX |
| OAP | UMP | DCZ | BSZ | SAX | KWX |
| PAP | IMP | NCZ | VSZ | HAX | WWX |
| ZAP | OMP | UCZ | DSZ | BAX | SWX |
| XAP | PMP | ICZ | NSZ | VAX | HWX |
| ACP | ZMP | OCZ | USZ | DAX | BWX |
| CCP | XMP | PCZ | ISZ | NAX | VWX |
| TCP | AOP | ZCZ | OSZ | UAX | DWX |
| GCP | COP | XCZ | PSZ | IAX | NWX |
| RCP | TOP | AGZ | ZSZ | OAX | UWX |
| YCP | GOP | CGZ | XSZ | PAX | IWX |
| MCP | ROP | TGZ | AXZ | ZAX | OWX |
| KCP | YOP | GGZ | CXZ | XAX | PWX |
| WCP | MOP | RGZ | TXZ | ATX | ZWX |
| SCP | KOP | YGZ | GXZ | CTX | XWX |
| HCP | WOP | MGZ | RXZ | TTX | AZX |
| BCP | SOP | KGZ | YXZ | GTX | CZX |
| VCP | HOP | WGZ | MXZ | RTX | TZX |
| DCP | BOP | SGZ | KXZ | YTX | GZX |
| NCP | VOP | HGZ | WXZ | MTX | RZX |
| UCP | DOP | BGZ | SXZ | KTX | YZX |
| ICP | NOP | VGZ | HXZ | WTX | MZX |
| OCP | UOP | DGZ | BXZ | STX | KZX |
| PCP | IOP | NGZ | VXZ | HTX | WZX |

| SZX |
| --- |
| HZX |
| BZX |
| VZX |
| DZX |
| NZX |
| UZX |
| IZX |
| OZX |
| PZX |
| ZZX |
| XZX |

Supplementary Table S2

Codon table without degenerative sequence used for address encoding.

| 0 | ACA | 12 | AAC | 24 | AAT | 36 | AAG |
| --- | --- | --- | --- | --- | --- | --- | --- |
| 1 | CCA | 13 | CAC | 25 | CAT | 37 | CAG |
| 2 | TCA | 14 | TAC | 26 | TAT | 38 | TAG |
| 3 | GCA | 15 | GAC | 27 | GAT | 39 | GAG |
| 4 | ATA | 16 | ATC | 28 | ACT | 40 | ACG |
| 5 | CTA | 17 | CTC | 29 | CCT | 41 | CCG |
| 6 | TTA | 18 | TTC | 30 | TCT | 42 | TCG |
| 7 | GTA | 19 | GTC | 31 | GCT | 43 | GCG |
| 8 | AGA | 20 | AGC | 32 | AGT | 44 | ATG |
| 9 | CGA | 21 | CGC | 33 | CGT | 45 | CTG |
| 10 | TGA | 22 | TGC | 34 | TGT | 46 | TTG |
| 11 | GGA | 23 | GGC | 35 | GGT | 47 | GTG |

Supplementary Table S3

Codon table with degenerative sequence W and S, used for data encoding.

| **Data** | **Codon** | **Data** | **Codon** | **Data** | **Codon** | **Data** | **Codon** |
| --- | --- | --- | --- | --- | --- | --- | --- |
| 1 | ACA | 33 | TTC | 65 | WGT | 97 | ACW |
| 2 | CCA | 34 | GTC | 66 | SGT | 98 | CCW |
| 3 | TCA | 35 | WTC | 67 | AST | 99 | TCW |
| 4 | GCA | 36 | STC | 68 | CST | 100 | GCW |
| 5 | WCA | 37 | AGC | 69 | TST | 101 | WCW |
| 6 | SCA | 38 | CGC | 70 | GST | 102 | SCW |
| 7 | ATA | 39 | TGC | 71 | WST | 103 | AGW |
| 8 | CTA | 40 | GGC | 72 | SST | 104 | CGW |
| 9 | TTA | 41 | WGC | 73 | AAG | 105 | TGW |
| 10 | GTA | 42 | SGC | 74 | CAG | 106 | GGW |
| 11 | WTA | 43 | AWC | 75 | TAG | 107 | WGW |
| 12 | STA | 44 | CWC | 76 | GAG | 108 | SGW |
| 13 | AGA | 45 | TWC | 77 | WAG | 109 | ASW |
| 14 | CGA | 46 | GWC | 78 | SAG | 110 | CSW |
| 15 | TGA | 47 | WWC | 79 | ACG | 111 | TSW |
| 16 | GGA | 48 | SWC | 80 | CCG | 112 | GSW |
| 17 | WGA | 49 | AAT | 81 | TCG | 113 | WSW |
| 18 | SGA | 50 | CAT | 82 | GCG | 114 | SSW |
| 19 | ASA | 51 | TAT | 83 | WCG | 115 | AAS |
| 20 | CSA | 52 | GAT | 84 | SCG | 116 | CAS |
| 21 | TSA | 53 | WAT | 85 | ATG | 117 | TAS |
| 22 | GSA | 54 | SAT | 86 | CTG | 118 | GAS |
| 23 | WSA | 55 | ACT | 87 | TTG | 119 | WAS |
| 24 | SSA | 56 | CCT | 88 | GTG | 120 | SAS |
| 25 | AAC | 57 | TCT | 89 | WTG | 121 | ATS |
| 26 | CAC | 58 | GCT | 90 | STG | 122 | CTS |
| 27 | TAC | 59 | WCT | 91 | AWG | 123 | TTS |
| 28 | GAC | 60 | SCT | 92 | CWG | 124 | GTS |
| 29 | WAC | 61 | AGT | 93 | TWG | 125 | WTS |
| 30 | SAC | 62 | CGT | 94 | GWG | 126 | STS |
| 31 | ATC | 63 | TGT | 95 | WWG | 127 | AWS |
| 32 | CTC | 64 | GGT | 96 | SWG | 128 | CWS |

Supplementary Table S4

Number of NGS read that acquired from each step.

|  | 3.37 bits/character (85nt) | | 2 bits/character (160nt) | |
| --- | --- | --- | --- | --- |
| Before Assemble | 162707 | 100% | 5847136 | 100% |
| Assemble | 158260 | 97% | 5660429 | 97% |
| Length filter | 127082 | 78% | 2928269 | 50% |
| Heterogeneous reads | 26675 | 16% | 1083343 | 19% |

1. Goldman, N. *et al.* Towards practical, high-capacity, low-maintenance information storage in synthesized DNA. *Nature* **494,** 77–80 (2013).

2. Erlich, Y. & Zielinsk, D. DNA Fountain enables a robust and efficient storage architecture. *Science (80-. ).* 950–954 (2017).

3. Organick, L. *et al.* Random access in large-scale DNA data storage. *Nat. Biotechnol.* (2018). doi:10.1038/nbt.4079

4. Grass, R. N., Heckel, R., Puddu, M., Paunescu, D. & Stark, W. J. Robust chemical preservation of digital information on DNA in silica with error-correcting codes. *Angew. Chem. Int. Ed. Engl.* **54,** 2552–5 (2015).
